# The Traffic Light Approach: Indicators and Algorithms to Identify Covid-19 Epidemic Risk Across Italian Regions

**DOI:** 10.3389/fpubh.2021.650243

**Published:** 2021-03-16

**Authors:** Luca Paroni, Clelia D'Apice, Silvia Ussai, Benedetta Armocida, Beatrice Formenti, Lorenzo De Min, Eduardo Missoni

**Affiliations:** ^1^Saluteglobale.It Associazione di Promozione Sociale, Brescia, Italy; ^2^Istituto di Ricerche Farmacologiche Mario Negri Istituti di Ricovero e Cura a Carattere Scientifico, Milan, Italy; ^3^Department of Medicine and Surgery, University of Parma, Parma, Italy; ^4^Directorate General for Food and Health, European Commission, Brussels, Belgium; ^5^Division of Infectious Diseases, Aziende Socio Sanitarie Territoriali Spedali Civili of Brescia, Brescia, Italy; ^6^Center for Research on Health and Social Care Management (CERGAS), Bocconi University, Milan, Italy

**Keywords:** COVID-19, Italy, guideline, indicators, traffic light algorithms

## Abstract

With the beginning of the autumn-winter season, Italy experienced an increase of SARS-CoV-2 cases, requiring the Government to adopt new restrictive measures. The national surveillance system in place defines 21 key process and performance indicators addressing for each Region/Autonomous Province: (i) the monitoring capacity, (ii) the degree of diagnostic capability, investigation and contact tracing, and (iii) the characteristics of the transmission dynamics as well as the resilience of health services. Overall, the traffic light approach shows a collective effort by the Italian Government to define strategies to both contain the spread of COVID-19 and to minimize the economic and social impact of the epidemic. Nonetheless, on what principles color-labeled risk levels are assigned on a regional level, it remains rather unclear or difficult to track.

With the beginning of the autumn-winter season, Italy experienced an increase of SARS-CoV-2 cases, requiring the Government to adopt new restrictive measures (1). The national surveillance system has been implemented according to the Prime Ministerial Decree (DPCM) of November 3, 2020 (2), the previous strategic documents for monitoring for the second wave (3, 4) and the DPCM of April 26, 2020 (5). The latter document defines 21 key process and performance indicators addressing for each Region/Autonomous Province: (i) the monitoring capacity, (ii) the degree of diagnostic capability, investigation and contact tracing, and (iii) the characteristics of the transmission dynamics as well as the resilience of health services (Table 1) (5). Based on a weekly assessment, each region is assigned to different levels of risk (high, medium, and low), which are mapped using a “traffic light” color code (red, orange, and yellow). The criteria are compliant with the European Commission's decision to adopt a common color code to provide strategy to restrict free movement across the European Union (6) and with the WHO frameworks (7).

**Table 1:** List of the 21 Indicators as described in the DPCM of April 26.

**Section**	***N***	**Indicator**	**Threshold**	**Alert**	**Data source**
Monitoring capacity (quality indicators of surveillance systems with data collection at national level)	1.1	Number of symptomatic cases notified per month with known symptoms start date/Total of symptomatic cases notified to the national surveillance system in the same period	≥60% with a growing trend; a value ≥50% with a growing trend will be considered acceptable in the first 3 weeks from 4 May 2020	<50% in the first 3 weeks from 4 May 2020, then <60%	Integrated national surveillance system
	1.2	Number of cases notified per month with a history of hospital admission (in wards other than ICU) indicating the date of admission/Total cases with a history of hospital admission (in wards other than ICU) notified to the national surveillance system in the same period			
	1.3	Number of cases notified per month with history of transfer/ICU admission indicating the date of transfer or ICU admission/Total of cases with history of transfer/ICU admission reported to the national surveillance system in the same period			
	1.4	Number of cases notified per month in which the municipality of domicile and residence is reported/Total of cases notified to the surveillance system in the same period			
	1.5 (optional)	Number of checklists provided weekly to residential healthcare facilities	≥50% of the residential healthcare facilities in the Region/Autonomous Province with an improving trend	<50% of the residential healthcare facilities in the Region/Autonomous Province	Periodic weekly evaluation by the Regions and Autonomous Provinces—Complementary surveillance to be carried out if feasible
	1.6 (optional)	Number of residential healthcare facilities responding to the checklist weekly with at least one criticality observed	≤30% with an improving trend	>30%	
Ability to promptly test all suspected cases	2.1	% of positive swabs per month excluding, where possible, all screening activities and “re-testing” of the same subject, total and by macro-setting (territory, ER/Hospital, other)	Decreasing trend in hospital settings/ER; Stable or decreasing Positive predictive value (PPV)	Increasing trend in hospital settings/ER; PPV increasing	Periodic weekly evaluation
	2.2	Time between symptoms onset and diagnosis	Weekly median ≤5 days	Weekly median >5 days	ISS; COVID-19 Integrated Surveillance System
	2.3 (optional)	Time between symptoms onset and start of isolation	Weekly median ≤3 days	Weekly median >3 days	ISS; COVID-19 Integrated Surveillance System with the integration of this variable
Ability to guarantee adequate resources for contact-tracing, isolation and quarantine	2.4	Number, types of professional figures and time per person dedicated in each territory to contact-tracing	Number and types of dedicated professionals dedicated to each activity at local level progressively aligned to recommended European standards	Number and types of dedicated professionals at local level reported as inadequate on the basis of recommending standards at European level	Periodic report (monthly)
	2.5	Number, types of professional figures and time per person dedicated in each territorial service to the activities of sampling/sending to the reference laboratories and monitoring of close contacts and cases placed, respectively in quarantine and isolation			
	2.6	Number of confirmed cases in the Region for which an appropriate epidemiological investigation has been carried out with investigation of close contacts/total of new confirmed cases of infection	Improving trend with final target to 100%		
Transmission stability	3.1	Number of cases notified to civil protection system in the last 14 days	Number of cases with decreasing or stable weekly trend	Cases increasing in the last 5 days (% of weekly increase with standard thresholds to be used as an “information dashboard”). An increase in the number of cases is expected in the first 15–20 days after reopening. In this phase, the warnings from this indicator will be evaluated together with indicators 3.1 and 3.5 at the regional level	Ministry of Health
	3.2	Rt calculated on the basis of integrated ISS surveillance (two indicators will be used. One based on date of onset of symptoms and the other based hospitalization date)	Rt calculated on regional level and ≤1 in all Regions/Autonomous Province in phase 2A	Rt > 1 or not measurable	ISS database/Rt measured by Foundation Bruno Kessler
	3.3 (optional)	Number of weekly cases notified to sentinel COVID-net surveillance	Number of cases with decreasing or stable trend	Cases increasing in the last 5 days (% of weekly increase with standard thresholds to be used as an “information dashboard”). An increase in the number of cases is expected in the first 15–20 days after reopening. In this phase, the warnings from this indicator will be evaluated together with indicators 3.1 and 3.5 at the regional level	ISS; Sentinel COVID-net surveillance
	3.4	Number of cases by diagnosis date and symptom onset date reported to COVID-19 integrated surveillance per day	Decreasing or stable weekly trend	Cases increased in the last week. An increase in the number of cases is expected in the first 15–20 days after reopening. In this alert, this indicator will be evaluated jointly with indicators 3.1 and 3.5 at the regional level (% of weekly increase with standard thresholds to be used as “information dashboard”	ISS-COVID-19 integrated surveillance system
	3.5	Number of new transmission outbreaks (2 or more epidemiologically linked cases or an unexpected increase in the number of cases in a defined time and place)	Non-increase the number of active transmission outbreaks in the Region; Absence of transmission outbreaks on the regional territory for which a risk assessment was not quickly carried out and the opportunity to establish a sub-regional “red zone” evaluated	Evidence of new outbreaks in the last 7 days, particularly in nursing homes, retirement homes, hospitals or other places hosting vulnerable populations. Presence of new local outbreaks in the region requires an *ad-hoc* risk evaluation in order to evaluate if in the regional area there is a substantial and spread transmission which would require to return on phase 1	ISS-Monitoring of outbreaks and red zones with survey cards OR ISS-Integrated Surveillance (using as a variable the point of exposure and defying a local outbreak ID)
			≥90% of responding facilities report the absence of subjects with a confirmed diagnosis of COVID-19 (optional)	<90% of responding facilities report the absence of subjects with a confirmed diagnosis of COVID-19 (optional)	Surveillance via checklist of residential facilities (optional)/ Complementary surveillance to be carried out based on feasibility
			Non-increase the number of active transmission outbreaks in the Region	Evidence of new outbreaks in the last 7 days especially in nursing homes, retirement homes, hospitals or other places hosting vulnerable populations	ISS—Activation of the Italian Epidemic Intelligence Network
	3.6	Number of regional new cases of confirmed SARS-CoV-2 infection not associated with a known chain of transmission	If there are new outbreaks declared, the indicator can monitor the quality of the contact-tracing. If there are no outbreaks of transmission, the presence of cases not connected to transmission chains could be compatible with a scenario of low transmission in which only sporadic cases are observed (considering a circulation rate not measurable in paucisymptomatic subjects)	In presence of outbreaks, the presence of new cases of infection not traced to non-contagion chains requires an *ad hoc* risk assessment that defines whether there is a sustained and widespread transmission in the region, such as to require a return to phase 1	Periodic weekly evaluation
	3.7 (optional)	Number of accesses to the ER with ICD-9 classification compatible with syndromic frameworks attributable to COVID-19	For at least 80% of the ER part of the surveillance network in the Region/Autonomous Province, number of ER accesses with syndromes compatible with COVID-19 decreasing or stable	In 50% of ER part of the surveillance network in the Region/Autonomous Province, number of ER accesses with syndromes compatible with COVID-19 increasing	ER Syndromic supervision coordination to be defined
Non-overloaded health care services	3.8	Total Intensive Care beds (code 49) occupancy rate of COVID-19 patients	≤30%	>30%	Ministry of Health—Platform for daily detection of beds/Civil Protection system admissions data
	3.9	Total medical area beds occupancy rate of COVID-19 patients	≤40%	>40%	

The complete algorithm is presented in Figure 1. The overall risk assessment is based on 21 key process and performance indicators (Table 1). Either thresholds or comparative evaluation are defined and monitored on a weekly basis by each region as part of the national integrated surveillance system.

**Figure 1:**
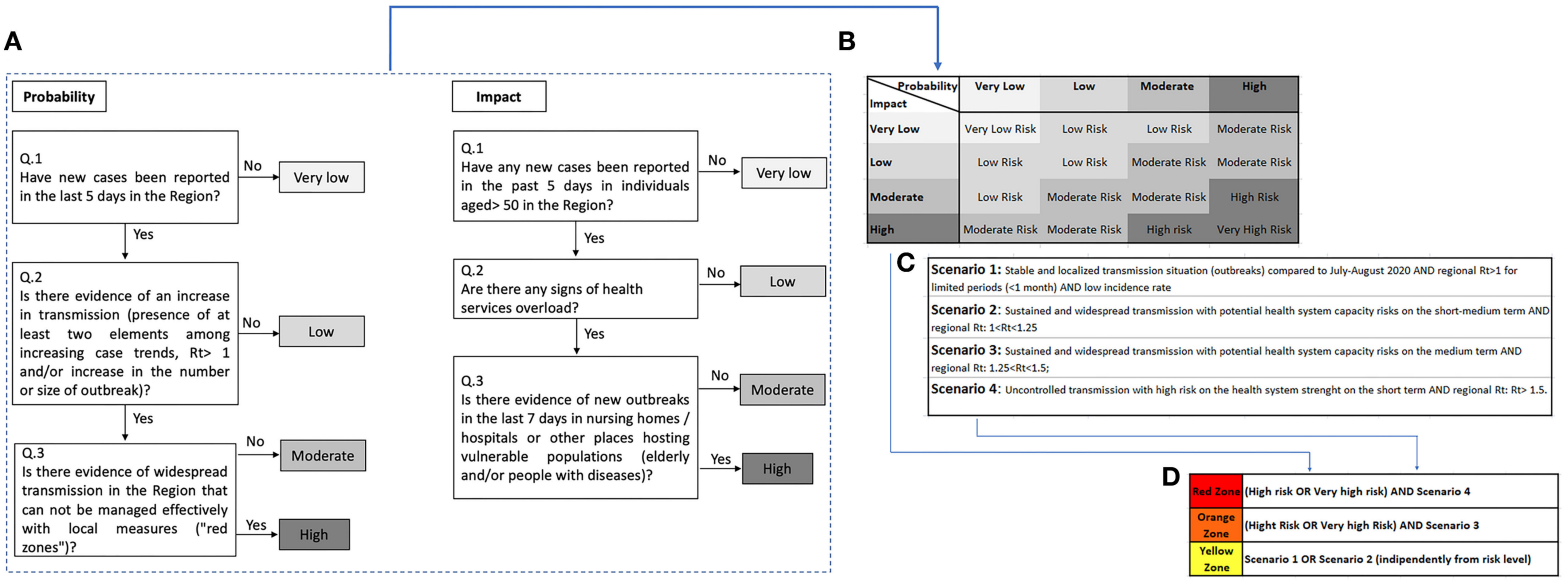
Complete algorithm. **(A)** Probability and Impact risk evaluation of COVID-19 pandemic from phase 1 to phase 2A in Italy based on the 21 indicators described in the DPCM from April 26. **(B)** Risk assessment matrix combination of probability and impact evaluation as described in the decree of the Ministry of Health. **(C)** Description of the four scenarios based on the strategic document developed by the Ministry of Health and ISS on the evolution of strategy and planning in the COVID-19 transition phase for the autumn-winter season (4). **(D)** Definition of the red, orange, and yellow areas based on the decree of the Ministry of health from November 3.

Indicators 1.1–1.6 are used for an initial assessment of the quality of the information collected. If a region has those indicators above threshold, it is automatically labeled as a high-risk region.

If a region complies with the threshold, two algorithms are used in order to evaluate the epidemic probability risk and epidemic impact risk (Figure 1A). Indicators used to evaluate the probability risk range from 3.1 to 3.7, while indicators used to evaluate the impact risk range from 3.1 to 3.9 (5). A risk assessment matrix (RAM) based on the probability and impact risk defines the risk level as: very low, low, moderate, high, and very high (Figure 1B). An additional classification of the level of risk has been defined assuming four possible scenarios based on the reproductive number (Rt) (Table 1—indicator 3.2) as described in Figure 1C (3).

For each region, the combination of RAM and the four possible scenarios defines the overall high, medium and low risk, which is color coded as red, orange, and yellow (2) (Figure 1D). A region is considered red when the RAM is equal to very high risk or high risk and complies with Scenario 4; orange when the RAM is equal to very high risk or high risk and complies with Scenario 3; and yellow when it is in Scenario 1 or Scenario 2, independently from the RAM results. From December 6 until December 12, the overall distribution of the Regions and Autonomous Provinces was as follows: 12 color-coded as yellow areas, eight color-coded as orange areas, and one as red area.

The risk assessment method outlined by the Italian Government is in line with the WHO and European Commission frameworks (6, 7). Although detailed indicators and the risk assessment process were defined, we draw attention to the following critical issues: (1) some indicators are not clearly outlined either in their meaning or in the proposed threshold (indicators 2.1, 2.4, 2.5, 2.6, 3.1–3.6); (2) the two algorithms of probability and impact risk assessment do not report which specific indicators are used to assess the four questions (Figure 1A); (3) overall, the information needed to define the entire algorithm is fragmented in different documents (2–5); (4) although the 21 indicators have been defined in the DPCM of April 26 (5), the related weekly reports started being described on a regional basis only from October 26 (8); (5) potential enforcement of the risk assessment algorithm by local regional regulation. As an example, the administration of the Abruzzo Region, which was on a Red risk level until December 5, decided to unilaterally self-declare on an orange risk level despite the Italian central Government did not allow the shift until December 12 (9).

The traffic light approach shows a collective effort by the Italian Government to define strategies to both contain the spread of COVID-19 and to minimize the economic and social impact of the epidemic. Nonetheless, on what principles color-labeled risk levels are assigned on a regional level, it remains rather unclear or difficult to track.

## Author Contributions

All authors listed have made a substantial, direct and intellectual contribution to the work, and approved it for publication.

## Conflict of Interest

The authors declare that the research was conducted in the absence of any commercial or financial relationships that could be construed as a potential conflict of interest.
